# EDR1 Physically Interacts with MKK4/MKK5 and Negatively Regulates a MAP Kinase Cascade to Modulate Plant Innate Immunity

**DOI:** 10.1371/journal.pgen.1004389

**Published:** 2014-05-15

**Authors:** Chunzhao Zhao, Haozhen Nie, Qiujing Shen, Shuqun Zhang, Wolfgang Lukowitz, Dingzhong Tang

**Affiliations:** 1 State Key Laboratory of Plant Cell and Chromosome Engineering, Institute of Genetics and Development Biology, Chinese Academy of Sciences, Beijing, China; 2 Graduate University of Chinese Academy of Sciences, Beijing, China; 3 Department of Biochemistry, University of Missouri, Columbia, Missouri, United States of America; 4 Department of Plant Biology, University of Georgia, Athens, Georgia, United States of America; Virginia Tech, United States of America

## Abstract

Mitogen-activated protein (MAP) kinase signaling cascades play important roles in the regulation of plant defense. The Raf-like MAP kinase kinase kinase (MAPKKK) EDR1 negatively regulates plant defense responses and cell death. However, how EDR1 functions, and whether it affects the regulation of MAPK cascades, are not well understood. Here, we showed that EDR1 negatively regulates the MKK4/MKK5-MPK3/MPK6 kinase cascade in Arabidopsis. We found that *edr1* mutants have highly activated MPK3/MPK6 kinase activity and higher levels of MPK3/MPK6 proteins than wild type. EDR1 physically interacts with MKK4 and MKK5, and this interaction requires the N-terminal domain of EDR1. EDR1 also negatively affects MKK4/MKK5 protein levels. In addition, the *mpk3*, *mkk4* and *mkk5* mutations suppress *edr1*-mediated resistance, and over-expression of *MKK4* or *MKK5* causes *edr1*-like resistance and mildew-induced cell death. Taken together, our data indicate that EDR1 physically associates with MKK4/MKK5 and negatively regulates the MAPK cascade to fine-tune plant innate immunity.

## Author Summary

Plant immunity must be tightly regulated, as over- or constitutive activation of plant defenses can cause detrimental effects, such as dwarf stature and enhanced cell death. EDR1, a Raf-like mitogen-activated protein kinase (MAPK) kinase kinase, negatively regulates defenses in Arabidopsis. The highly conserved MAPK cascades modulate diverse biological processes, including plant immunity. However, whether EDR1 affects the regulation of one of the MAPK pathways was not previously known. Here, we show that EDR1 physically associates with MKK4 and MKK5, two MAP kinase kinases, and negatively regulates the protein levels of MKK4, MKK5, MPK3 and MPK6. We further show that *edr1*-mediated disease resistance requires MKK4, MKK5 and MPK3 function. Over-expression of *MKK4* or *MKK5* in wild-type increased resistance to powdery mildew and caused mildew-induced cell death. Our study suggests that EDR1 negatively regulates defenses and directly modulates the MKK4/MKK5-MPK3/MPK6 cascade to fine-tune plant immunity.

## Introduction

Mitogen-activated protein kinase (MAPK) cascades are highly conserved signaling modules that control diverse signal transduction pathways in eukaryotes, including defenses against infection [1]. Activation of MAPK cascades is thought to be one of the earliest events in plant immunity [1]–[3]. For instance, treatment of Arabidopsis with a conserved 22-amino acid peptide fragment of bacterial flagellin, a pathogen-associated molecular pattern (PAMP), specifically recognized by the pattern recognition receptor FLS2, can trigger activation of MKK4/MKK5 and MPK3/MPK6 [4], [5], which subsequently promotes expression of the downstream target gene *FRK1* and activates plant defenses [5]. Other PAMPs, such as EF-Tu, chitin, harpin, oligogalacturonides and xylanase, also trigger the activation of MPK3 and/or MPK6 [6]–[9]. In addition, MPK3 and MPK6 regulate phytoalexin biosynthesis by activating the transcription factor WRKY33, which is required for resistance to necrotrophic fungal pathogens [10]–[12]; also, the full priming of stress responses in Arabidopsis requires MPK3 and MPK6 [13]. MKK4 and MKK5 function redundantly and act upstream of MPK3 and MPK6. Constitutive activation of MKK4 and MKK5 in Arabidopsis leads to HR-like cell death associated with the generation of reactive oxygen species [14]. Plants expressing active forms of MKK4 and MKK5 have enhanced resistance to *Pseudomonas syringae* and *Botrytis cinerea* [5].

Plant basal defenses require the MKK4/MKK5-MPK3/MPK6 kinase cascade. To effectively invade plants, bacterial pathogens block PAMP-induced defenses with effectors, such as AvrPto, AvrPtoB, HopAI1, and HopF2, that directly repress MKK4/MKK5 and MPK3/MPK6 activities or inhibit the components that act upstream of MAP kinase cascades [15]–[20]. In Arabidopsis, five phosphatases have been reported that regulate MPK3 and MPK6 kinase activity by dephosphorylation. For instance, the PP2C-type phosphatase AP2C1 inactivates stress-induced kinase activity of MPK4 and MPK6 [21]; PP2C5 also regulates ABA-mediated activation of MPK3 and MPK6 [22]. Also, MKP1 and PTP1 phosphatases repress salicylic acid biosynthesis and SNC1-mediated responses by inactivating MPK3 and MPK6 [23]. Also, MKP2 interacts with and dephosphorylates MPK3 and MPK6 to regulate oxidative stress and plant defense responses [24], [25]. However, the mechanisms of the negative regulation of MKK4/MKK5 in *Arabidopsis* remain unclear.

The Raf-like MAPK kinase kinase (MAPKKK) EDR1 functions as a negative regulator of plant defense. For example, *edr1* mutants have enhanced resistance to pathogens including powdery mildew fungus, bacteria and oomycetes [26], [27]. The *edr1* mutants also show enhanced ethylene-induced senescence [28]. EDR1 protein consists of an N-terminal functionally unknown domain and a C-terminal kinase domain. The kinase activity of EDR1 has been demonstrated *in vitro* [29]. *KEEP ON GOING* (*KEG*) encodes a protein containing RING E3 ligase domain, kinase domain, ankyrin repeats and HERC2-like repeats [30] and the recessive missense mutant *keg-4* suppresses the phenotype of *edr1* mutants. KEG could directly interact with EDR1 and recruit EDR1 to the trans-Golgi network/early endosome [31].

In Arabidopsis, the EDR1 homolog CTR1 (Constitutive Triple Response 1), a Raf-like MAPKKK [29], plays an essential role in the negative regulation of ethylene signaling [32]. CTR1 and the MKK9-MPK3/MPK6 cascade antagonistically regulate ethylene responses [33]. However, whether EDR1 affects the regulation of one specific MAPK cascade pathway remains unknown. The *edr1* mutants are constitutively primed for salicylic acid-inducible defenses and enhanced callose deposition, which may be mediated by the regulation of MPK3 and MPK6 in Arabidopsis [13], [34]. However, the molecular mechanisms leading to enhanced resistance and cell death in *edr1* are still not well understood. Here, we report that EDR1 negatively regulates the MKK4/MKK5-MPK3/MPK6 kinase cascade pathway through direct interaction with MKK4 and MKK5 to modulate plant defense and cell death.

## Results

### EDR1 negatively affects MPK3 and MPK6 protein levels and kinase activity

**Figure 1:**
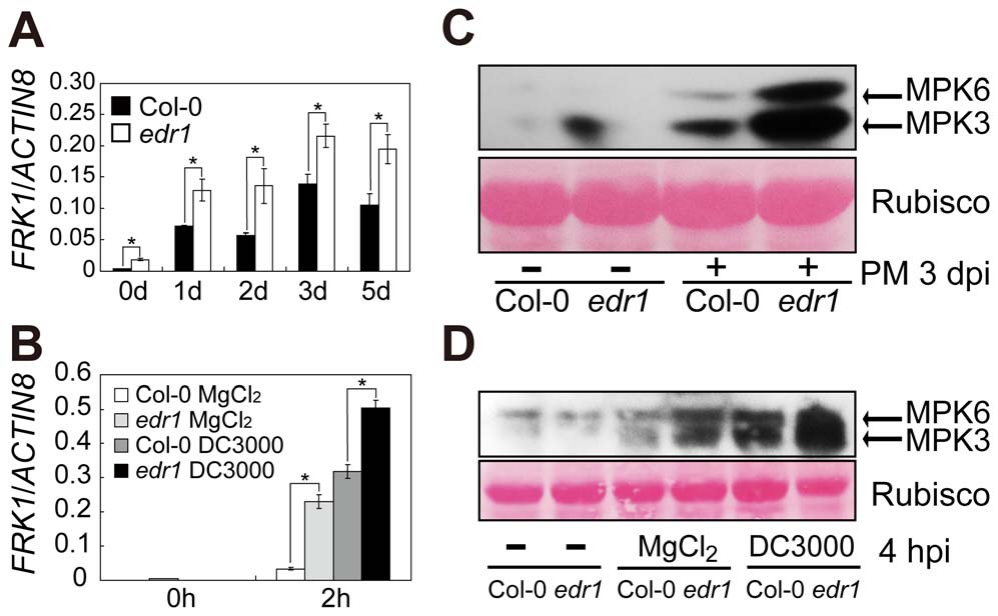
EDR1 negatively regulates the kinase activity of MPK3 and MPK6. (**A–B**) The transcript accumulation of *FRK1* was measured by quantitative real-time RT-PCR. Leaves were collected for RNA isolation at different time points after infection with *G. cichoracearum* (**A**) or *Pto* DC3000 (in 10 mM MgCl_2_) (**B**). Error bars represent the standard deviation of three biological replicates. Asterisks indicate statistically significant differences (P<0.05, Student's *t*-test). (**C–D**) The plants were infected with *G. cichoracearum* (**C**) and *Pto* DC3000 (**D**), respectively. Immunoblotting was performed using an anti-phospho-p44/42 MAPK (Thr202/Tyr204) (anti-pTEpY) antibody. The large subunit of Rubisco is shown as a protein loading control. The experiment was repeated at least three times with similar results. PM: powdery mildew infection.

EDR1 belongs to the MAPKKK family, but a mechanistic link to MAP kinase cascades has remained elusive. After treatment with benzothiadiazole (BTH), the activity of MPK3 and MPK6 was higher in *edr1* mutants than in Col-0 [13]. In addition, large-scale co-expression data analysis [35], [36] showed that *MKP1*, which is involved in the negative regulation of MPK3/MPK6 by dephosphorylation in Arabidopsis [23], is one of the top-ranking genes co-expressed with *EDR1* (Figure S1). Based on these results, we hypothesized that EDR1 may function as a negative regulator of the MPK3/MPK6 kinase cascade pathway in pathogen responses. Consistent with this notion, the accumulation of *FRK1* transcript, a target of the MPK3/MPK6 cascade [37], was significantly higher in *edr1* compared to wild-type upon infection with powdery mildew *Golovinomyces cichoracearum* or *Pseudomonas syringae* pv. *tomato* (*Pto*) DC3000 (Figure 1A and 1B).

Along with up-regulation of the *FRK1* gene, MPK3 and MPK6 kinase activation also increased in *edr1* compared to wild-type after infection by powdery mildew or *Pto* DC3000 (Figure 1C and 1D). These data indicate that EDR1 negatively affects the MPK3/MPK6 cascade.

**Figure 2:**
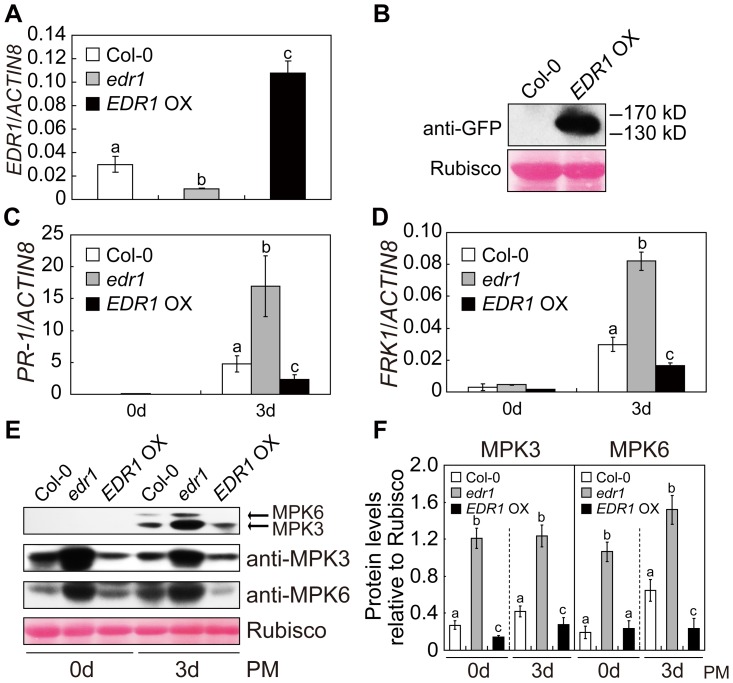
Over-expression of *EDR1* reduced the kinase activity and protein levels of MPK3 and MPK6. (**A**) The transcript accumulation of *EDR1* was examined by quantitative real-time RT-PCR for wild-type Col-0, *edr1* and *EDR1* over-expressing plants. *ACTIN8* was used as an internal control. Error bars represent the standard deviation of three biological replicates. Different letters represent statistically significant differences (P<0.05, one-way ANOVA). (**B**) EDR1-GFP is properly expressed in *EDR1* over-expressing plants. Immunoblotting was performed using anti-GFP antibody in four-week-old plants. The large subunit of Rubisco is shown as a protein loading control. (**C–D**) The transcript accumulation of *PR-1* (**C**) or *FRK1* (**D**) was examined by quantitative real-time RT-PCR. Leaves from Col-0, *edr1* and *EDR1* over-expressing plants after infection by powdery mildew for 0 d and 3 d were collected for RNA isolation. Error bars represent the standard deviation of three biological replicates. Different letters represent statistically significant differences (P<0.05, one-way ANOVA). (**E**) Col-0, *edr1* and *EDR1* over-expressing plants were infected by *G. cichoracearum*. Immunoblot was performed using anti-pTEpY, anti-MPK3 and anti-MPK6 antibodies, as indicated. The large subunit of Rubisco is shown as a protein loading control. The experiment was repeated three times with similar results. PM: powdery mildew infection. (**F**) The protein bands of MPK3 and MPK6, as well as Rubisco, were quantified with ImageJ. The protein levels of MPK3 and MPK6 in each sample were evaluated by comparing to Rubisco. The error bars represent the standard deviation of three biological replicates. Different letters represent statistically significant differences (P<0.05, one-way ANOVA). PM: powdery mildew infection.

To further examine the role of EDR1 in the MPK3/MPK6 pathway, we over-expressed *EDR1* in Arabidopsis and investigated whether over-expression of *EDR1* reduces MPK3 and MPK6 kinase activity. Previously, an attempt to over-express EDR1 with cauliflower mosaic virus (CaMV) 35S promoter driven *EDR1* coding sequence (CDS) was not successful [29]. Therefore, we generated over-expression lines by introducing GFP-tagged *EDR1* genomic sequence under its native promoter into the *edr1* mutant. This construct complemented all *edr1* mutant phenotypes, including *edr1*-mediated enhanced resistance to powdery mildew and enhanced cell death (Figure S2A, S2B and S2C). For further analysis, we selected one transgenic line with three-fold up-regulation of *EDR1* and with properly expressed EDR1-GFP protein, as demonstrated by immunoblotting assays (Figure 2A and 2B). The *EDR1* over-expressing plants showed enhanced susceptibility to powdery mildew, as significantly more spores of *G. cichoracearum* were produced after 5 days inoculation compared to wild-type (Figure S2D). However, the growth of *Hyaloperonospora arabidopsidis* Noco2 on the *EDR1* over-expressing plants and wild-type was not significantly different (Figure S2E). The *EDR1* over-expressing plants showed delayed ethylene-induced senescence compared to wild-type (Figure S2F and S2G). Furthermore, the *EDR1* over-expressing plants showed lower levels of *PR-1* and *FRK1* expression and lower MPK3 and MPK6 kinase activity than wild-type upon infection by powdery mildew (Figure 2C, 2D and 2E).

We then examined the accumulation of MPK3 and MPK6 proteins in *edr1* and *EDR1* over-expressing plants using specific anti-MPK3 and anti-MPK6 antibodies. Compared to wild-type, the protein levels of MPK3 and MPK6 were significantly higher in the *edr1* mutants, but lower in *EDR1* over-expressing plants, even in normal conditions without infection by pathogens (Figure 2E and 2F). To further demonstrate that EDR1 negatively regulates the protein level of MPK3, we transiently expressed *MPK3* alone or with *EDR1* in *Nicotiana benthamiana*. The level of MPK3 was significantly lower when we co-expressed *MPK3* with *EDR1* (Figure S3A). Transient expression in *N*. *benthamiana* further showed that expression of the N-terminal presumptive regulatory domain of EDR1 (1–657 aa), but not the C-terminal kinase domain (658–933 aa), was sufficient to suppress the accumulation of MPK3 (Figure S3B and S3C). In contrast, the accumulation of *MPK3* and *MPK6* transcripts was not significantly affected in the *edr1* mutants infected with powdery mildew (Figure S4A and S4B), indicating that the regulation of MPK3 and MPK6 by EDR1 mainly functions at the protein level, not the mRNA level.

As EDR1 plays a negative role in plant defense, we also examined abundance of EDR1 protein during pathogen infection. Leaves of *EDR1-Flag* transgenic plants were infected by *G. cichoracearum*, and the proteins were extracted 0 d, 2 d, and 5 d after inoculation, respectively. We found that levels of EDR1 protein significantly declined upon powdery mildew infection (Figure S5), consistent with the negative role of EDR1 in defense responses.

A missense mutation (*keg-4*) in the E3 ubiquitin ligase KEG suppresses all *edr1*-associated phenotypes [30]. To examine whether the *keg-4* mutation also counteracts the elevated activation of the MPK3 kinase cascade pathway, we examined the protein level of MPK3 in the *edr1 keg-4* double mutant before and after infection with *Pto* DC3000. In the *edr1 keg-4* mutant, the MPK3 protein level was significantly lower than in the *edr1* mutants, indicating that the *keg-4* mutation also suppressed increased accumulation of MPK3 protein in the *edr1* mutants (Figure S6).

### Loss of MPK3 suppresses the *edr1* phenotype

**Figure 3:**
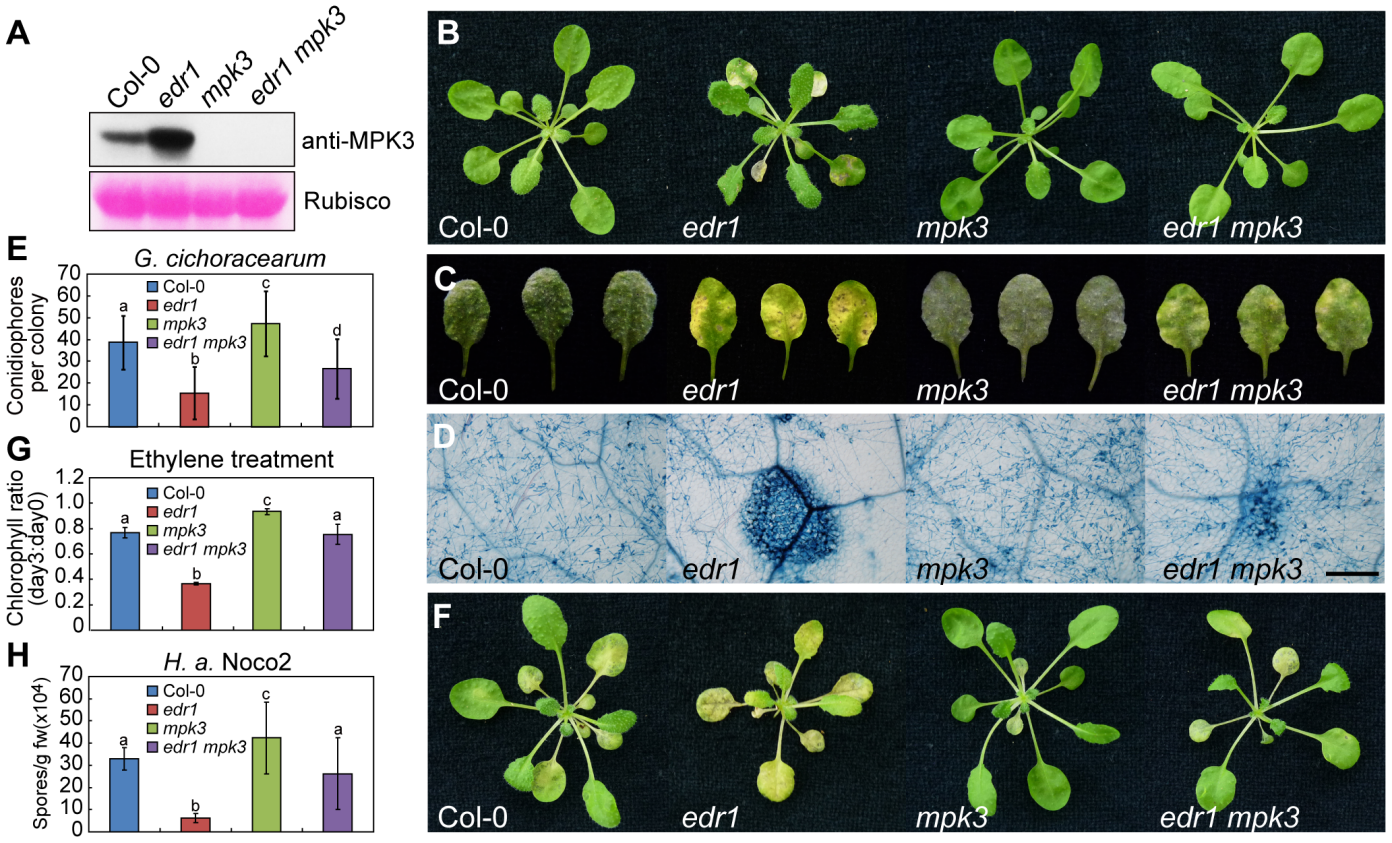
The *mpk3-1* mutation suppressed the *edr1* phenotype. (**A**) Immunoblot for Col-0, *edr1*, *mpk3-1* and *edr1 mpk3-1* was performed using specific anti-MPK3 antibody. The large subunit of Rubisco is shown as a protein loading control. (**B**) Col-0, *edr1*, *mpk3-1* and *edr1 mpk3-1* were grown in the greenhouse at 22°C and a 9 h light/15 h dark cycle. Pictures were taken after 5 weeks growth. (**C**) Plants were infected with *G. cichoracearum*. Pictures were taken at 7 dpi. (**D**) Powdery mildew infected leaves at 7 dpi were stained by trypan blue. Bar = 0.3 mm. (**E**) Quantification of fungal growth by counting the number of conidiophores per colony at 5 dpi. At least 30 colonies were counted for each sample. Error bars represent the standard deviation. Different letters represent statistically significant differences (P<0.05, one-way ANOVA). (**F**) Four-week-old plants of Col-0, *edr1*, *mpk3-1* and *edr1 mpk3-1* were treated with ethylene (100 µL/L) for three days in a sealed chamber. Pictures were taken after 3 days. (**G**) Chlorophyll content was measured in wild-type Col-0, *edr1*, *mpk3-1* and *edr1 mpk3-1* before and after treatment of ethylene (3 days). The ratio of chlorophyll content at day 3 to day 0 was calculated for each sample. Error bars represent the standard deviation of ten plants. Different letters represent statistically significant differences (P<0.05, one-way ANOVA). (**H**) Three-week-old Col-0, *edr1*, *mpk3-1* and *edr1 mpk3-1* plants were infected by *H. a.* Noco2. Spores were counted at 7 dpi. Error bars represent the standard deviation of three biological replicates. Different letters represent statistically significant differences (P<0.05, one-way ANOVA). The above experiments were repeated three times with similar results.

To further examine the role of MPK3/MPK6 in *edr1*-mediated defense, we conducted crosses to make double mutants of *edr1* and *mpk3-1* or *mpk6-3* (Figure 3A and S7A). The *mpk3-1* mutation suppressed the early senescence and spontaneous cell death associated with *edr1* mutants (Figure 3B); it also partially suppressed the resistance of *edr1* to powdery mildew and *H. a.* Noco2. In addition, the *mpk3-1* mutation counteracted *edr1*-mediated enhanced ethylene-induced senescence, restoring it to wild-type levels (Figure 3C–3H). In contrast, *mpk6-3* did not suppress these *edr1* phenotypes (). These observations indicate that *edr1*-mediated resistance to pathogens and cell death requires MPK3, but not MPK6.

To further examine the functions of MPK3 and MPK6 in plant defense, we transformed wild-type plants with *MPK3* and *MPK6* expressed under the control of the 35S promoter. Although we obtained a number of *MPK3* transgenic lines, none of them showed higher *MPK3* expression, suggesting that Arabidopsis may not tolerate the over-expression of *MPK3*. We did obtain transgenic plants with higher expression levels of *MPK6* (Figure S8A and S8B), but these *MPK6* over-expressing plants showed wild-type-like responses to powdery mildew (Figure S8C). This suggests that the high level of MPK6 in *edr1* mutants does not contribute to *edr1*-mediated resistance to powdery mildew, consistent with the observation that the *mpk6* mutation did not affect *edr1* phenotypes.

### EDR1 interacts with MKK4 and MKK5

**Figure 4:**
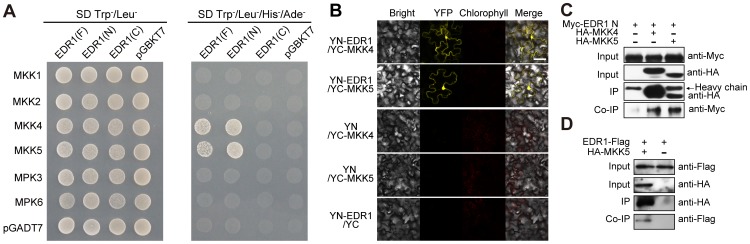
EDR1 interacts with MKK4 and MKK5. (**A**) *EDR1* full length (F), *EDR1* N-terminal domain (N) and *EDR1* C-terminal domain (C) were fused to the Gal4 DNA binding domain (BD). *MKK1*, *MKK2*, *MKK4*, *MKK5*, *MPK3* and *MPK6* were fused to the Gal4 transactivation domain (AD). Different pairs of constructs were cotransformed into yeast isolate AH109 to test the interaction. 10 µL suspension (OD_600_ = 0.5) of each cotransformant was dropped on the synthetic dropout (SD) medium lacking Leu and Trp (left) and SD medium lacking Ade, His, Leu and Trp (right), respectively. Pictures were taken after 2 days incubation. (**B**) YFP^YN^-fused *EDR1* and YFP^YC^-fused *MKK4*/*MKK5* were co-expressed in *N*. *benthamiana*. YFP fluorescence was detected by confocal microscopy. Cotransformants of YFP^YN^-EDR1 and YFP^YC^, YFP^YN^ and YFP^YC^-MKK4, or YFP^YN^ and YFP^YC^-MKK5 were used as controls. Bar = 50 µm. (**C**) *EDR1* N-terminal domain was expressed alone or co-expressed with *MKK4* and *MKK5* in *N*. *benthamiana*. Proteins were extracted after 48 h, and subjected to immunoprecipitation by anti-HA antibody, followed by immunoblotting using anti-Myc and anti-HA antibodies, respectively. (**D**) *EDR1-Flag* transgenic plants and *EDR1-Flag*/*HA-MKK5* double transgenic plants were used for co-IP. The proteins were analyzed by immunoblotting using anti-Flag or anti-HA antibody, respectively. The above experiments were repeated three times with similar results.

To examine whether EDR1 directly regulates the MPK3/6 kinase cascade, we used a yeast two-hybrid assay to test for potential interactions of EDR1 with MPK3 or MPK6, or the upstream MAP kinases MKK4 and MKK5 [5], [38]. We found that EDR1 interacts with MKK4 and MKK5 (Figure 4A), but not with MPK3 or MPK6. As a control, we also assayed the interaction of EDR1 with the well-studied MAP kinase kinases MKK1 and MKK2. EDR1 did not interact with either MKK1 or MKK2 (Figure 4A), indicating that EDR1 interacts specifically with MKK4 and MKK5. To determine which domain of EDR1 is responsible for the interaction with MKK4 and MKK5, we tested the EDR1 N-terminal domain and C-terminal kinase domain and found that the EDR1 N-terminal domain is responsible for the interaction with MKK4 and MKK5 (Figure 4A).

We used several complementary approaches to examine whether EDR1 also interacts with MKK4 and MKK5 *in vivo*. First, we performed bimolecular fluorescence complementation (BiFC) assays by transiently co-expressing YFP^YN^-fused *EDR1* and YFP^YC^-fused *MKK4* or *MKK5*, in *N*. *benthamiana*. We detected YFP fluorescence only in cells co-expressing YFP^YN^-EDR1 with YFP^YC^-MKK4 or YFP^YN^-EDR1 with YFP^YC^-MKK5, but not in the negative controls (Figure 4B). Second, to confirm the association of EDR1 and MKK4/MKK5, we performed co-immunoprecipitation (co-IP) assays and found that MKK4 and MKK5 immunoprecipitated the EDR1 N-terminal domain upon transient expression in *N. benthamiana* (Figure 4C). Furthermore, MKK5 and EDR1 could also be precipitated from stable transgenic Arabidopsis plants expressing both EDR1-Flag and MKK5-HA (Figure 4D). Third and finally, we examined whether EDR1 and MKK4/MKK5 co-localize in Arabidopsis. We crossed plants harboring GFP-tagged MKK4 or MKK5 transgenes with plants harboring a Cherry-tagged EDR1 transgene, and imaged GFP and Cherry fluorescence by confocal microscopy in the F1 generation. We found that EDR1 and MKK4/MKK5 co-localize in the cytoplasm and, partially, in the nucleus (Figure S9A and S9B).

### EDR1 negatively regulates the levels of MKK4 and MKK5

**Figure 5:**
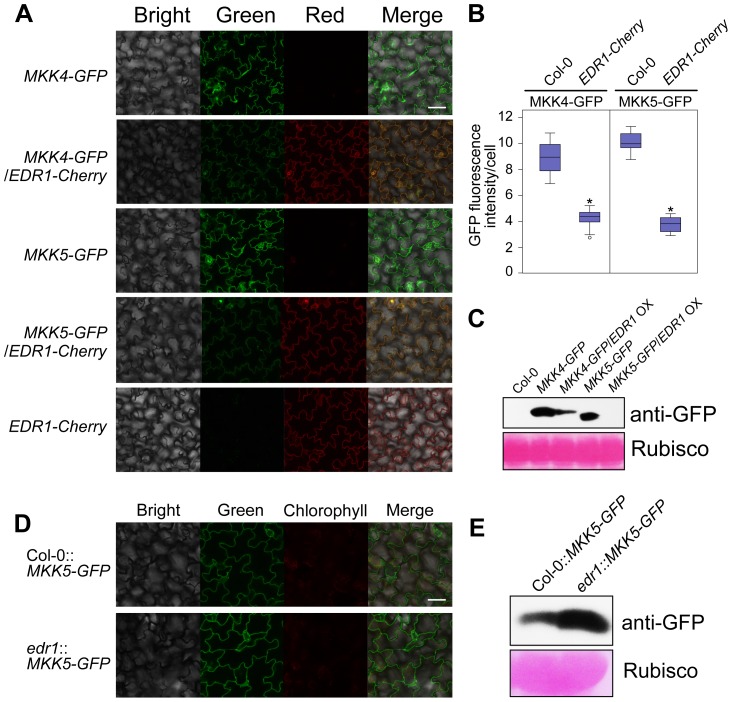
EDR1 regulates the protein levels of MKK4 and MKK5. (**A**) GFP and Cherry fluorescence of seedlings of transgenic plants that express MKK4-GFP or MKK5-GFP alone or with EDR1-Cherry, was detected by confocal microscopy using the same parameters. Bar = 50 µm. (**B**) The GFP fluorescence intensity was quantified by using ImageJ software. 30 cells from 10 independent leaves of each transgenic plant were used for the quantification of the intensity of GFP fluorescence. The results are shown as a box plot graph. Asterisks represent statistically significant differences (P<0.05, Student's *t*-test). (**C**) Immunoblot was performed for each sample using anti-GFP antibody. The large subunit of Rubisco is shown as a protein loading control. (**D**) GFP fluorescence of seedlings of transgenic plants Col-0::*MKK5-GFP* and *edr1*::*MKK5-GFP* was detected by confocal microscopy using the same parameters. Bar = 50 µm. (**E**) Immunoblot was performed for Col-0::*MKK5-GFP* and *edr1*::*MKK5-GFP* using anti-GFP antibody. The large subunit of Rubisco is shown as a protein loading control.

Since EDR1 physically associates with MKK4 and MKK5, we reasoned that EDR1 may also affect the protein levels of MKK4 and MKK5. As MKK4 and MKK5 specific antibodies are not available, we used the same transgenic lines described above to examine the accumulation of GFP-tagged MKK4 or MKK5 in plants with higher or lower levels of EDR1. Analyzing more than 30 individuals for each transgene combination by confocal microscopy, we found that plants expressing both *MKK4 g-GFP* and *EDR1 g-Cherry*, or both *MKK5 g-GFP* and *EDR1 g-Cherry* showed less-intense GFP fluorescence than transgenic plants expressing *MKK4 g-GFP* or *MKK5 g-GFP* alone (Figure 5A and 5B). In contrast, the intensity of Cherry fluorescence was not affected. Immunoblotting assays confirmed that protein levels of MKK4 and MKK5 were significantly lower in the presence of an *EDR1* transgene (Figure 5C). Furthermore, we combined the *MKK5 g-GFP* transgene with the *edr1* mutation; as expected, MKK5-GFP accumulated to higher levels in the *edr1* background compared to wild-type on the basis of both GFP fluorescence intensity and immunoblotting assays (Figure 5D and 5E). These data indicate that EDR1 negatively affects the protein levels of MKK4 and MKK5.

### *edr1*-mediated resistance to powdery mildew requires MKK4 and MKK5

**Figure 6:**
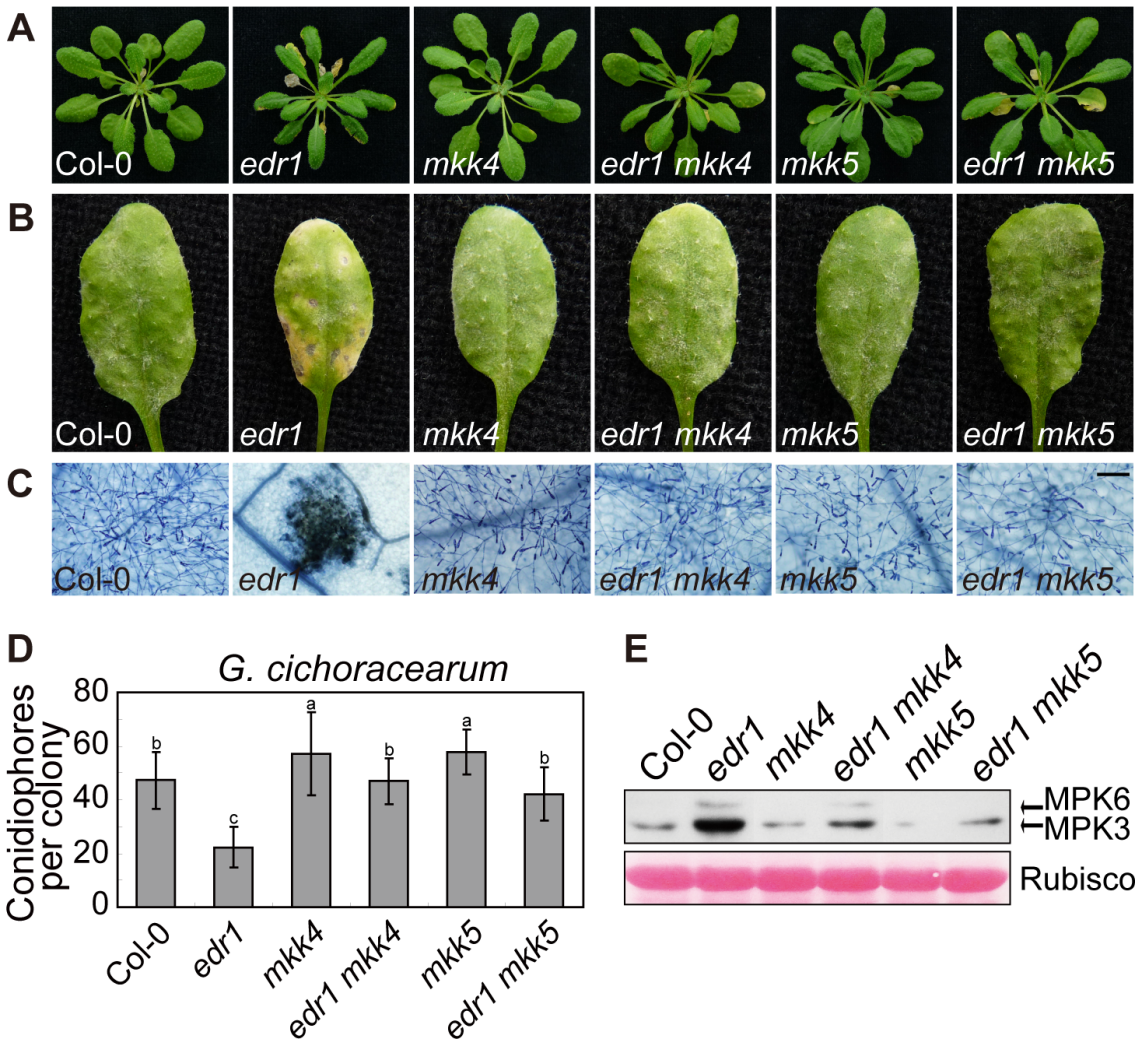
*mkk4* and *mkk5* suppress *edr1*-mediated resistance to powdery mildew and cell death. (**A**) Col-0, *edr1*, *mkk4*, *edr1 mkk4*, *mkk5* and *edr1 mkk5* were grown in the greenhouse at 22°C and a 9 h light/15 h dark regime. Pictures were taken after 5 weeks of growth. (**B**) Plants were infected by *G. cichoracearum*. Pictures were taken at 7 dpi. (**C**) Powdery mildew infected leaves at 7 dpi were stained by trypan blue. Bar = 0.1 mm. (**D**) Fungal growth was assessed by counting the number of conidiophores at 5 dpi. At least 30 colonies were counted for each sample. Error bars represent the standard deviation. Different letters represent statistically significant differences (P<0.05, one-way ANOVA). (**E**) Plants were infected by *G. cichoracearum* for 3 days. Immunoblot was performed using anti-pTEpY antibody. The large subunit of Rubisco is shown as a protein loading control. The experiment was repeated three times with similar results.

To investigate whether the enhanced powdery mildew resistance of *edr1* mutants is due to the elevated protein levels of MKK4 and MKK5, we generated double mutant combinations of *edr1* and loss-of-function alleles of *MKK4* or *MKK5*. The *mkk4–18* mutation results in the substitution of proline-240, a conserved position in the catalytic site, to serine. *yda-2*, a strong loss-of-function allele of the MAPKKK YDA, harbors the same exchange in the homologous position [39], suggesting that this amino acid is important for protein function. The mutation in *mkk5–18* leads to premature termination of translation (R72stop). Both the *mkk4* and *mkk5* mutation suppressed *edr1*-mediated early senescence and cell death after 5 weeks growth (Figure 6A). Following inoculation with *G. cichoracearum*, both the *mkk4* and *mkk5* mutation counteracted the resistance conferred by *edr1*, such that susceptibility of double mutants was close to wild-type (Figure 6B, 6C and 6D). In addition, the leaves of *mkk4* and *mkk5* single mutants produced more spores of powdery mildew than wild-type leaves, indicating that both mutants show higher susceptibility to powdery mildew. This enhanced susceptibility phenotype could be complemented by introducing a genomic DNA fragment spanning the MKK4 or MKK5 locus, respectively (Figure S10), indicating that MKK4 and MKK5 are involved in disease resistance to powdery mildew. Finally, the activity of the MPK3/MPK6 kinases was lower in *edr1 mkk4* and *edr1 mkk5* than in *edr1* after infection by powdery mildew (Figure 6E).

To further study the functions of MKK4 and MKK5 in plant defense, we overexpressed *MKK4* and *MKK5* in wild-type (Figure S11A, S11B and S11C); plants over-expressing *MKK4* or *MKK5* showed enhanced resistance to powdery mildew and displayed *edr1*-like spontaneous cell death (Figure S11D and S11E). In summary, these results indicate that MKK4 and MKK5 play a positive role in powdery mildew resistance, and implicate the elevated protein levels of MKK4 and MKK5 as the main mechanism leading to pathogen resistance in the *edr1* mutant.

## Discussion

The essential MKK4/MKK5-MPK3/MPK6 kinase cascade transduces extracellular stimuli in many different response pathways. Activation of this cascade must be tightly controlled, as inappropriate activation of MAPKs can inhibit growth or even cause lethality. For instance, in Arabidopsis, constitutively activated MKK4 and MKK5 cause an accelerated cell death phenotype [14]. Over-expression of MPK3 may be lethal for the plant, as we (and others) have found it difficult to obtain *MPK3* over-expressing plants ([13], [40] and this study). Therefore, tight regulation of MKK4/MKK5 and MPK3/MPK6 kinase activity and protein accumulation seems essential for survival and adaptation to environmental challenges. The kinase activity of MPK3/MPK6 can be repressed through dephosphorylation mediated by several different phosphatases [21]–[25]. Modulation of MKK4 and MKK5 protein levels by EDR1 could be another mechanism for fine-tuning the activity of the MKK4/MKK5-MPK3/MPK6 kinase cascade in response to pathogen attack.

How EDR1 affects accumulation of MKK4 and MKK5 protein remains unresolved. Our study showed that the N-terminal regulatory domain of EDR1 interacts with MKK4 and MKK5, and negatively affects MKK4 and MKK5 accumulation. One possible mechanism for this effect may be that EDR1 acts as a scaffold protein that keeps MKK4 and MKK5 in a catalytically inactive state. While MKK4 and MKK5 are not active, the plants would deploy a feedback regulation mechanism to keep MKK4 and MKK5 protein levels low in order to avoid detrimental spontaneous activation of defense responses. Low levels of MKK4 and MKK5 protein in turn would lead to low accumulation of MPK3 and MPK6 protein. An alternative explanation for our finding is that EDR1 affects the stability of MKK4 and MKK5 directly, perhaps through interactions with the 26S proteasome degradation machinery. Consistent with this possibility, EDR1 has been reported to associate with the E3 ubiquitin ligase KEG [30], [31], a component of the SCF ligase complex, and that mutations in KEG suppress *edr1*-mediated resistance. Furthermore, we recently showed that *edr1*-mediated defense responses require RPN1a, a subunit of the 26S proteasome [41], suggesting that 26S proteasome degradation machinery is involved in the EDR1 pathway.

Although MKK4 and MKK5 are commonly considered, by implication, to be important components of plant immunity pathways, little genetic support for this notion has been reported. Here, we identified *mkk4* and *mkk5* loss-of-function mutants, and showed that they display enhanced susceptibility to powdery mildew. We also showed that *edr1*-mediated resistance requires MKK4 and MKK5 function, providing direct genetic evidence that MKK4 and MKK5 are key players in plant immunity responses. The *mkk4* and *mkk5* alleles described in this study will be valuable tools for dissecting the role of the MKK4/5 mediated MAPK cascade in biotic and abiotic stresses.

The MKK4 and MKK5 proteins are considered to be functionally equivalent [1], [42]. Interestingly, our study revealed that single mutation of either MKK4 or MKK5 significantly affect *edr1*-mediated defense. Consistent with this observation, both *mkk4* and *mkk5* single mutant showed enhanced susceptibility to powdery mildew, indicating that loss-of-function of either one of these two genes causes a defect in immune responses. Dosage effects may be a possible explanation for these observations. In this scenario, the accumulation of MKK4 and MKK5 protein is limiting, such that the activity of both genes is required for normal immune responses. This view is supported by our finding that over-expression of either *MKK4* or *MKK5* enhanced resistance to powdery mildew and resulted in *edr1*-like cell death. However, the possibility that MKK4 and MKK5 have non-identical roles in plant immunity has not been rigorously tested and cannot be dismissed at this time.

Arabidopsis has more than sixty MAPKKKs, which can be divided into two subfamilies, including 12 MEKK1-like kinases and approximately 50 Raf-like kinases [43]. Several members of the MEKK1 family have been shown to function as MAPKKKs upstream of MAPKKs in MAP kinase cascades [1]; in contrast, no evidence has implicated any of the Raf-like kinases as canonical MAPKKKs. EDR1 and CTR1 are the only two well-characterized Raf-like kinases in plants. CTR1 directly regulates EIN2 by phosphorylation; EIN2, in turn, inactivates EIN3/EIL1-dependent ethylene responses. This indicates that CTR1, at least in this context, does not function as a MAPKKK [44], [45]. Here, we show that the N-terminus of EDR1 associates with MKK4/MKK5 and negatively affects the accumulation of MKK4/5 protein, suggesting that EDR1 does not act as a MAPKKK either. Instead, EDR1 may fine-tune responses to biotic and abiotic stresses by controlling this MAP kinase cascade.

Several proteins that were originally identified as negative regulators of plant immunity, such as LSD1 and ACD11, have on closer analysis turned out not be true negative regulators, as loss-of-function alleles lead to inappropriate activation of NBS-LRR proteins, suggesting that LSD1 and ACD11 may be guarded pathogen targets [46], [47]. However, EDR1 appears to be different from those proteins, as over-expression of EDR1 leads to enhanced susceptibility and lower activation of MAPK cascade, indicating that EDR1 could serve as a negative regulator of defense by repressing the MAPK pathway. In the absence of pathogen, EDR1 inactivates the MAPK pathways; however, upon pathogen infection, plants quickly activate defenses, possibly by de-repression of the inhibition of MAPK pathways by EDR1.

In conclusion, we show that EDR1 directly associates with MKK4 and MKK5, and negatively affects protein levels of MKK4, MKK5, MPK3 and MPK6, which may represent an important mechanism that fine-tunes plant defense responses.

## Materials and Methods

### Plant materials and growth conditions

The *Arabidopsis thaliana mpk3-1* (SALK_151594) and *mpk6-3* (SALK_127507) mutants were obtained from the Arabidopsis Stock Center (ABRC; Ohio State University; Columbus, OH). The homozygous T-DNA insertion mutants were confirmed by PCR. The *edr1* mutant was described previously [26]. Mutations in *MKK4* and *MKK5* were isolated by the Arabidopsis TILLING facility from EMS-mutagenized *Col er-105* plants [48]. The *mkk4–18* allele has a substitution at a conserved position of the catalytic domain (proline-240 to serine; CCT to TCT). The *mkk5–18* allele has a premature stop codon (arginine-72 to opal; CGA to TGA). The *er-105* mutation was removed by crossing with Col-0. Plants were grown in the growth room at 20–22°C as described previously [49]. For molecular complementation of *mkk4* and *mkk5* mutants, genomic DNA fragments spanning both loci (MKK4: 2.3 kb total, including approximately 0.6 kb upstream and downstream of the coding sequence; MKK5: 2.5 kb total, including approximately 0.6 kb upstream and 0.9 kb downstream of the coding sequence) were introduced into *mkk4* or *mkk5* mutants, respectively. Three independent lines from each transformation were selected for further analyses.

### Pathogen infection and ethylene assay

For powdery mildew infection, four-week-old plants were inoculated with *G. cichoracearum* strain UCSC1 as described previously [50]. To quantify the resistance, the conidiophores per colony were counted at 5 dpi. *P. syringae* pv. *tomato* (*Pto*) DC3000 infection assay and ethylene treatment assays were performed as described previously [51]. For *H. a.* Noco2 infection assay, three-week-old plants were used for infection [52].

### Construction of plasmids

To generate *EDR1* genomic-GFP and *EDR1* genomic-Cherry constructs, the *EDR1* genomic sequence including 1077 bp upstream of the ATG start codon was amplified (TOYOBO), and cloned into the pDONR207 ENTRY vector, and then into pMDC107 and pMDC163-Cherry destination vectors, respectively, using the Gateway cloning system (Invitrogen). The *MKK4* and *MKK5* genomic-GFP constructs were constructed using a similar strategy. For HA tagged *MKK4*, *MKK5*, *MPK3* and *MPK6* constructs, the coding sequence (CDS) of each gene was amplified and cloned into the pDNOR207 ENTRY vector and then into the pEarleyGate 201 destination vector.

For the yeast two-hybrid assay, the CDS sequences of full-length *EDR1*, *EDR1* N-terminal domain and *EDR1* C-terminal domain were amplified and ligated into vector pGBKT-7, and the CDS sequences of *MKK1*, *MKK2*, *MKK4*, *MKK5*, *MPK3* and *MPK6* were amplified and ligated into vector pGADT-7. For the BiFC assay, 35S-YN-EDR1, 35S-YC-MKK4 and 35S-YC-MKK5 were constructed according to the procedure described previously [53].

### Immunoblotting and co-immunoprecipitation analysis

For protein extraction, leaves collected from *Nicotiana benthamiana* or Arabidopsis were ground in liquid nitrogen and the proteins were extracted using native extraction buffer (50 mM Tris-MES pH 8.0, 0.5 M sucrose, 1 mM MgCl_2_, 10 mM EDTA, 5 mM DTT and protease inhibitor cocktail S8830 (Sigma)). The total extraction was mixed well and centrifuged at 12000 rpm and 4°C for 30 min. The suspension was transferred to a new tube for further analysis. For immunoblotting, proteins were separated by SDS-PAGE (10% acrylamide gel) and transferred to PVDF membrane (Millipore) by electro-transfer at 80 V for 90 min. The membrane was blocked in 1× TBS buffer containing 5% skim milk powder and further incubated with primary antibody and secondary antibody. Finally the bands were detected using chemiluminescent HRP substrate (Millipore). For the co-IP assay, 1 ml protein extraction was incubated with 3 µL HA antibody for 4 hrs and then 40 µL Protein G (50% slurry, Millipore) was added to the cell lysates for another 2 h to capture the immunocomplex. The mixture was washed 3 times with cold PBS buffer containing 0.1% IGEPAL CA-630 (Sigma) and once with cold PBS buffer. Finally the agarose beads were resuspended in 50 µL 2× Laemmli sample buffer and 20 µL of supernatant was used for immunoblot. Antibodies used for immunoblotting were as follows: anti-HA antibody (1∶3000, H3663, Sigma), anti-GFP antibody (1∶2000, 632569, Clontech), anti-Myc antibody (1∶2000, M20002M, Abmart), anti-Flag antibody (1∶2000, F1804, Sigma), anti-MPK3 antibody (1∶10000, Sigma), anti-MPK6 antibody (1∶10000, Sigma) and anti–Phospho-p44/p42 MAPK (anti-pTEpY) (1∶2000, Cell Signaling Technology).

### Yeast two-hybrid assay

For yeast two-hybrid assays, two constructs (pGBKT-7 and pGADT-7 containing the respective genes) were cotransformed into yeast AH109 strain (Clontech). The transformants were plated on synthetic dropout (SD) agar plates containing adenine and histidine for selection. A single colony for each transformant was incubated in the SD liquid medium containing adenine and histidine for 2 days. Subsequently, the concentration of each suspension was measured and diluted to OD 0.5, and 10 µL of each dilution was dropped on the selection medium SD for 2 days incubation.

### Fluorescence assay

For BiFC assay, YFP^N^ and YFP^C^ fused proteins or YFP^N^ and YFP^C^ empty vector were co-transformed into *N. benthamiana* by *Agrobacterium*-mediated transformation. 48 hours later, the YFP fluorescence was detected by confocal microscopy.

For the colocalization assay, *EDR1* pro-*EDR1* genomic sequence-Cherry construct (*EDR1 g-Cherry*), *MKK4* pro-*MKK4* genomic sequence-GFP construct (*MKK4 g-GFP*) and *MKK5* pro-*MKK5* genomic sequence-GFP construct (*MKK5 g-GFP*) were transformed into wild-type, respectively. Then the copy number of the *MKK4 g-GFP*, *MKK5 g-GFP* and *EDR1 g-Cherry* expression constructs was determined, and the plants contained a single locus of the transgene were selected. And then, *MKK4 g-GFP*, *MKK5 g-GFP* transgenic plants were crossed with *EDR1 g-Cherry* transgenic plants, respectively, to generate double transgenic plants. The fluorescence of GFP and Cherry was observed by confocal microscopy.

For the comparison of the intensity of fluorescence, *MKK4 g-GFP*/*EDR1 g-Cherry* or *MKK5 g-GFP*/*EDR1 g-Cherry* double transgenic plants mentioned above were selected to compare with the corresponding *MKK4 g-GFP* or *MKK5 g-GFP* transgenic line. The transgenes are all homozygous, so the plants have the same genetic background, except for the specific transgene of interest. Identical parameters, including same laser strength and same pinhole, were applied for all samples. ImageJ software (http://rsb.info.nih.gov/ij) was used to quantify the intensity of GFP fluorescence.

## Supporting Information

Figure S1 Bioinformatics analysis of co-expression of EDR1 and MKP1. (**A**) Genes co-expressed with *EDR1* were analyzed by ATTED-II. The graph shows the network of co-expressed genes around *EDR1*. (**B**) Co-correlation analysis according to the Arabidopsis Co-expression Tool (ACT). *MPK1* and *EDR1* (highlighted in red) are both located at the top right, indicating that their expression is highly correlated.
(PDF)

Figure S2 Over-expression of EDR1 led to enhanced susceptibility to pathogens. (**A**) The *EDR1 g*-*GFP* construct complemented *edr1*-mediated early senescence and cell death. The *edr1* mutant developed lesions and became chlorotic after 5 weeks growth, but no lesions or chlorosis were observed in Col-0 and *EDR1* transgenic plants. (**B**) Plants were infected by *G. cichoracearum*. Pictures were taken at 7 dpi. (**C**) Powdery mildew infected leaves were stained by trypan blue at 7 dpi. Pictures were taken by microscopy. Bar = 0.2 mm. (**D**) Fungal growth was quantified by counting the number of conidiophores per colony at 5 dpi. At least 30 colonies were counted for each sample. Error bars represent the standard deviation. Different letters represent statistically significant differences (P<0.05, one-way ANOVA). (**E**) Col-0, *edr1* and *EDR1* over-expressing plants were infected by *H. a.* Noco2. The spores were counted at 7 dpi. Different letters represent statistically significant differences (P<0.05, one-way ANOVA). (**F**) Four-week-old plants were treated with ethylene (100 µL/L) in a sealed chamber. Pictures were taken after 3 days. (**G**) The chlorophyll contents of Col-0, *edr1* and *EDR1* over-expressing plants were measured before and after treatment with ethylene (3 days). The ratio of chlorophyll content at day 3 to day 0 was calculated for each sample. Error bars represent the standard deviation of six plants. Different letters represent statistically significant differences (P<0.05, one-way ANOVA).
(PDF)

Figure S3 Transient expression of *MPK3* in *N*. *benthamiana*. Transient expression of *MPK3* alone or co-expression with *EDR1* full length (**A**), *EDR1* N-terminal domain (**B**) and *EDR1* C-terminal domain (**C**), respectively, in *N*. *benthamiana*. The proteins were extracted for immunoblot using anti-HA antibody and anti-GFP antibody. The experiment was repeated twice with similar results. The large subunit of Rubisco is shown as a protein loading control.
(PDF)

Figure S4 mRNA levels of *MPK3* and *MPK6*. Col-0 and *edr1* mutants were inoculated with *G. cichoracearum*. The inoculated leaves were collected at 0 d, 1 d, 2 d, 3 d and 5 d for RNA isolation, and quantitative real-time RT-PCRs were performed using *MPK3* (**A**) and *MPK6* (**B**) specific primers. *ACTIN8* was used as internal control. Error bars represent the standard deviation of three biological replicates. PM: powdery mildew infection.
(PDF)

Figure S5 EDR1 protein level is decreased after pathogen infection. *EDR1-Flag* transgenic plants were infected by *G. cichoracearum* for 0 d, 2 d and 5 d, respectively. The infected leaves were collected at each time point and the proteins were extracted for immunoblotting using anti-Flag antibody. The experiment was repeated for three times with similar results. The large subunit of Rubisco is shown as a protein loading control.
(PDF)

Figure S6 The *keg-4* mutation inhibits the elevated protein levels of MPK3 in *edr1.* Col-0, *edr1* and *edr1 keg-4* mutants were infected with *Pto* DC3000. Proteins were extracted and immunoblots were performed using anti-MPK3 antibody. The large subunit of Rubisco is shown as a protein loading control.
(PDF)

Figure S7 Analysis of *edr1 mpk6-3* double mutant. (**A**) Immunoblotting was performed for Col-0, *edr1*, *mpk6-3* and *edr1 mpk6-3* using specific anti-MPK6 antibody. The large subunit of Rubisco is shown as a protein loading control. (**B**) Col-0, *edr1*, *mpk6-3* and *edr1 mpk6-3* were grown in the greenhouse at 22°C and a 9 h light/15 h dark cycle. Pictures were taken after 5 weeks growth. (**C**) Col-0, *edr1*, *mpk6-3* and *edr1 mpk6-3* were infected by *G. cichoracearum*. Pictures were taken at 7 dpi.
(PDF)

Figure S8 Over-expression of *MPK6* in *Arabidopsis*. (**A**) Quantitative real-time RT-PCR was performed for Col-0 and *MPK6* transgenic plants using *MPK6* specific primers. *ACTIN8* was used as the internal control. Error bars represent the standard deviation of three biological replicates. (**B**) Immunoblot was performed for *MPK6* transgenic plants using anti-HA antibody. The large subunit of Rubisco is shown as a protein loading control. (**C**) Col-0 and over-expression line Col-0::*MPK6-2* were inoculated with *G. cichoracearum*. Pictures (Top) were taken at 7 dpi. The inoculated leaves were stained by trypan blue (Bottom). Bar = 0.2 mm.
(PDF)

Figure S9 Co-localization of EDR1 and MKK4/MKK5 in *Arabidopsis*. The subcellular localization of MKK4-GFP and EDR1-Cherry (**A**) or MKK5-GFP and EDR1-Cherry (**B**) in leaves and roots were examined by confocal microscopy. The middle image represents higher magnification of the upper picture. Bar = 50 µm.
(PDF)

Figure S10 A genomic clone of *MKK4* or *MKK5* complemented enhanced powdery mildew susceptibility in *mkk4* or *mkk5*. (**A**) Plants were infected by *G. cichoracearum*, and pictures were taken at 7 dpi. (**B**) Leaves infected with *G. cichoracearum* at 7 dpi were stained by trypan blue. Bar = 0.1 mm. (**C**) Fungal growth was assessed at 5 dpi by counting the number of conidiophores per colony. Error bars represent the standard deviation (n>30). Statistically significant differences were indicated by different letters (P<0.05, one-way ANOVA). Three independent transgenic lines of *mkk4* (containing a *MKK4* genomic clone, MKK4 line 5, 7 and 9) and *mkk5* (containing a *MKK5* genomic clone, MKK5 line 39, 53 and 54) were included.
(PDF)

Figure S11 Over-expression of *MKK4* and *MKK5* led to *edr1*-like enhanced resistance to powdery mildew and mildew-induced cell death. (**A**) Immunoblot assay was performed for *MKK4* and *MKK5* transgenic plants using anti-HA antibody. Rubisco is shown as a protein loading control. (**B**–**C**) Quantitative real-time RT-PCRs were performed for *MKK4* and *MKK5* transgenic plants using *MKK4* and *MKK5* specific primers, respectively. *ACTIN8* was used as an internal control. Error bars represent the standard deviation of three biological replicates. (**D**) Col-0, *edr1* and transgenic plants *35S::MKK4* and *35S::MKK5* were infected with *G. cichoracearum*. Pictures were taken at 7 dpi. (**E**) Powdery mildew infected leaves at 7 dpi were stained by trypan blue. Pictures were taken by microscopy. Bar = 0.2 mm. (**F**) Fungal growth was assessed by counting the number of conidiophores per colony at 5 dpi. At least 30 colonies were counted for each sample. Error bars represent the standard deviation. Different letters represent statistically significant differences (P<0.05, one-way ANOVA).
(PDF)
