# Supplementary figures and images for: EDR1 Physically Interacts with MKK4/MKK5 and Negatively Regulates a MAP Kinase Cascade to Modulate Plant Innate Immunity
Source: PLoS Genet. 2014 May 15;10(5):e1004389. doi: 10.1371/journal.pgen.1004389 (PMC4022593; doi:10.1371/journal.pgen.1004389)

Figure S1

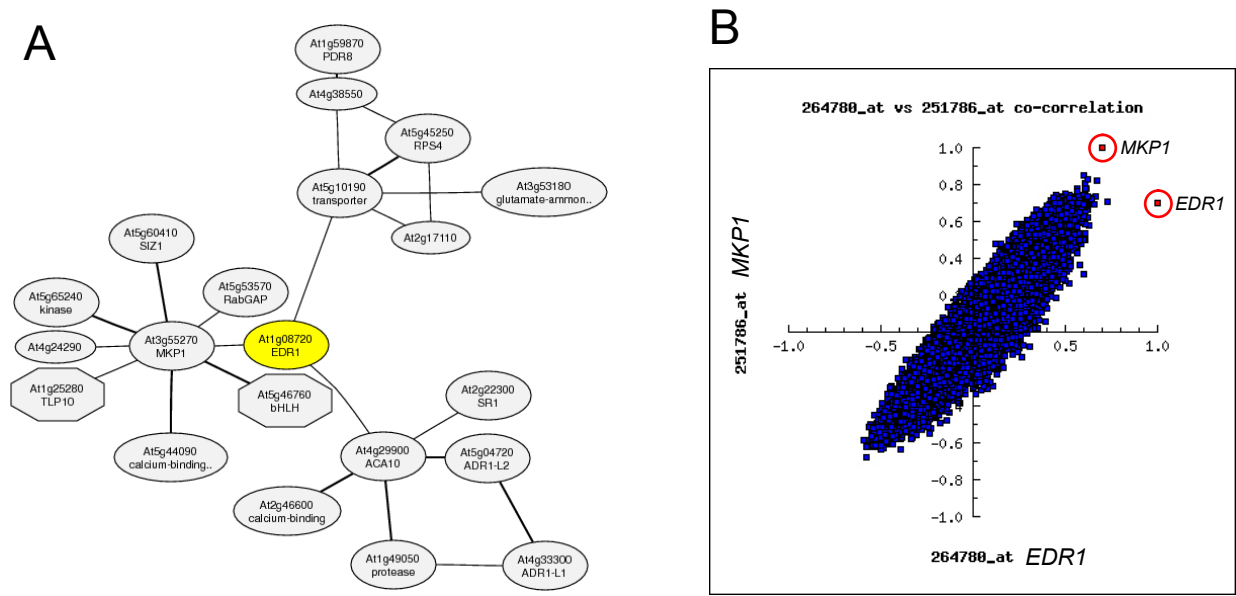

Supplement: Figure S1 — Bioinformatics analysis of co-expression of EDR1 and MKP1. (A) Genes co-expressed with EDR1 were analyzed by ATTED-II. The graph shows the network of co-expressed genes around EDR1. (B) Co-correlation analysis according to the Arabidopsis Co-expression Tool (ACT). MPK1 and EDR1 (highlighted in red) are both located at the top right, indicating that their expression is highly correlated. (PDF) [file pgen.1004389.s001.pdf]

Figure S2

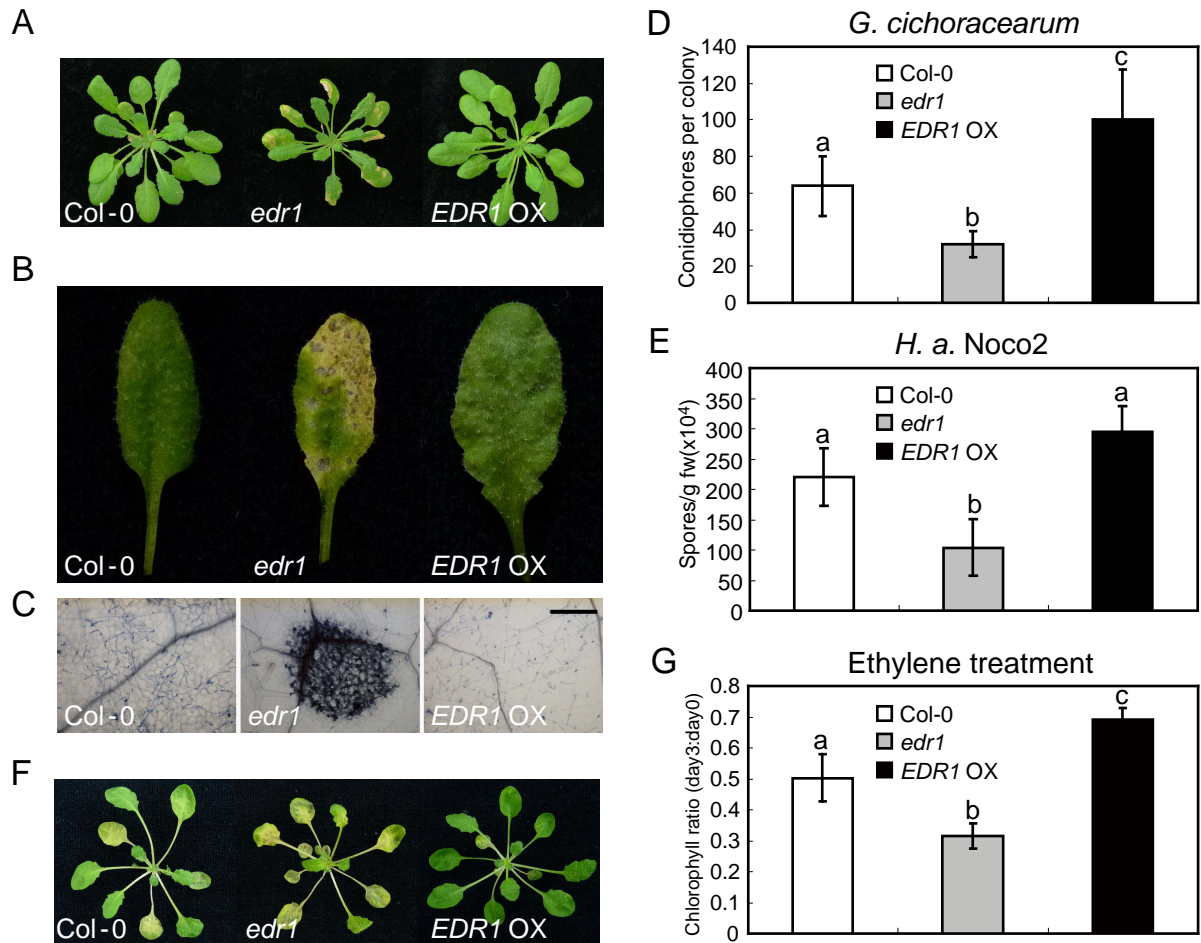

Supplement: Figure S2 — Over-expression of EDR1 led to enhanced susceptibility to pathogens. (A) The EDR1 g-GFP construct complemented edr1-mediated early senescence and cell death. The edr1 mutant developed lesions and became chlorotic after 5 weeks growth, but no lesions or chlorosis were observed in Col-0 and EDR1 transgenic plants. (B) Plants were infected by G. cichoracearum. Pictures were taken at 7 dpi. (C) Powdery mildew infected leaves were stained by trypan blue at 7 dpi. Pictures were taken by microscopy. Bar = 0.2 mm. (D) Fungal growth was quantified by counting the number of conidiophores per colony at 5 dpi. At least 30 colonies were counted for each sample. Error bars represent the standard deviation. Different letters represent statistically significant differences (P<0.05, one-way ANOVA). (E) Col-0, edr1 and EDR1 over-expressing plants were infected by H. a. Noco2. The spores were counted at 7 dpi. Different letters represent statistically significant differences (P<0.05, one-way ANOVA). (F) Four-week-old plants were treated with ethylene (100 µL/L) in a sealed chamber. Pictures were taken after 3 days. (G) The chlorophyll contents of Col-0, edr1 and EDR1 over-expressing plants were measured before and after treatment with ethylene (3 days). The ratio of chlorophyll content at day 3 to day 0 was calculated for each sample. Error bars represent the standard deviation of six plants. Different letters represent statistically significant differences (P<0.05, one-way ANOVA). (PDF) [file pgen.1004389.s002.pdf]

Figure S3

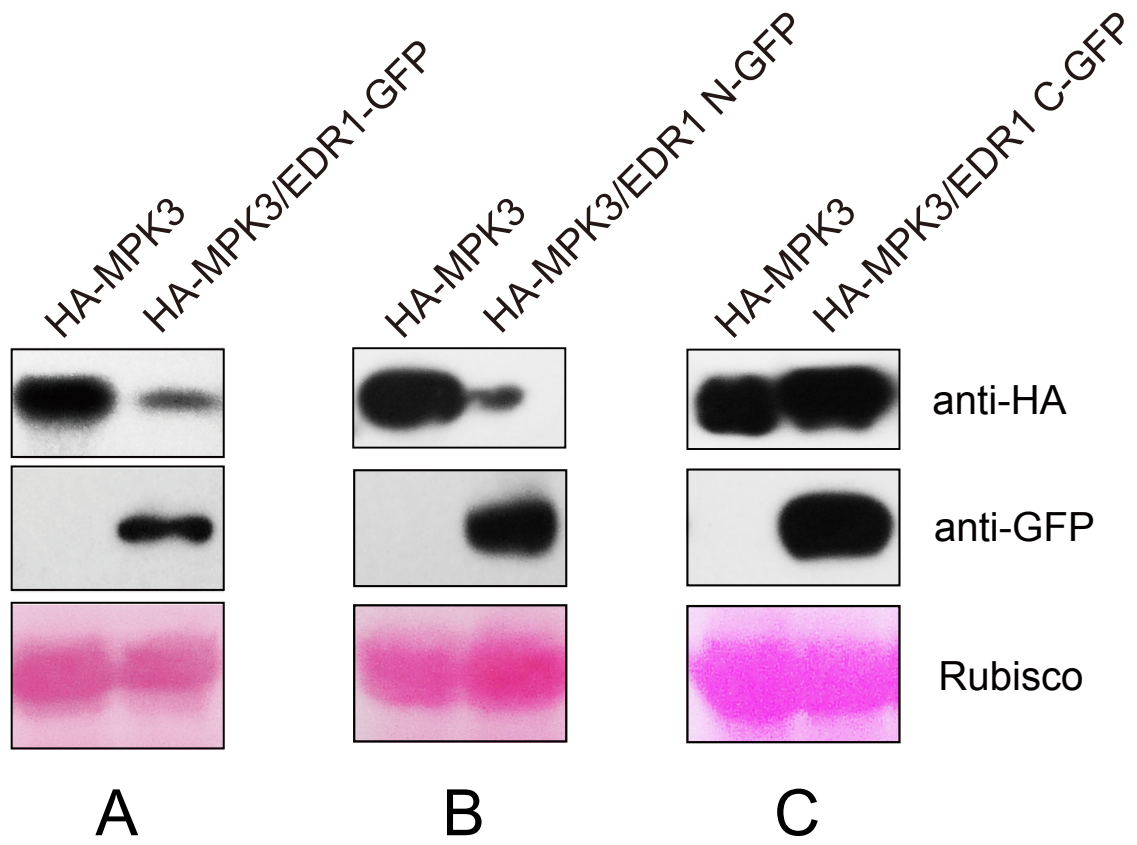

Supplement: Figure S3 — Transient expression of MPK3 in N. benthamiana. Transient expression of MPK3 alone or co-expression with EDR1 full length (A), EDR1 N-terminal domain (B) and EDR1 C-terminal domain (C), respectively, in N. benthamiana. The proteins were extracted for immunoblot using anti-HA antibody and anti-GFP antibody. The experiment was repeated twice with similar results. The large subunit of Rubisco is shown as a protein loading control. (PDF) [file pgen.1004389.s003.pdf]

Figure S4

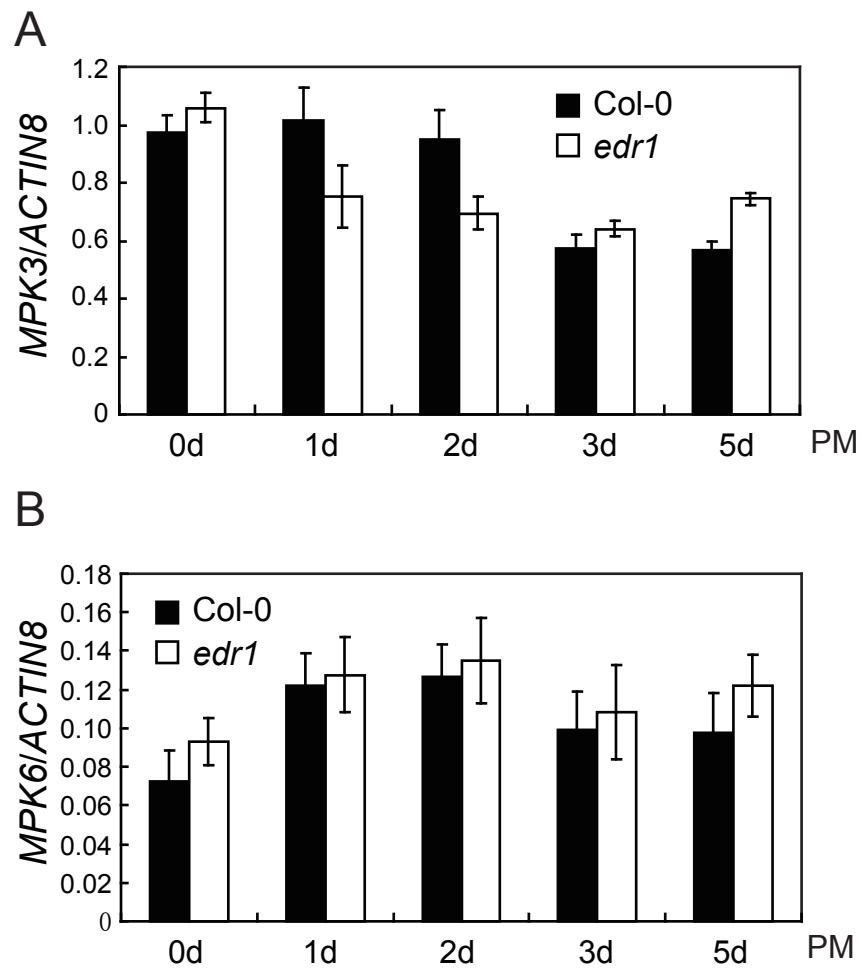

Supplement: Figure S4 — mRNA levels of MPK3 and MPK6. Col-0 and edr1 mutants were inoculated with G. cichoracearum. The inoculated leaves were collected at 0 d, 1 d, 2 d, 3 d and 5 d for RNA isolation, and quantitative real-time RT-PCRs were performed using MPK3 (A) and MPK6 (B) specific primers. ACTIN8 was used as internal control. Error bars represent the standard deviation of three biological replicates. PM: powdery mildew infection. (PDF) [file pgen.1004389.s004.pdf]

Figure S5

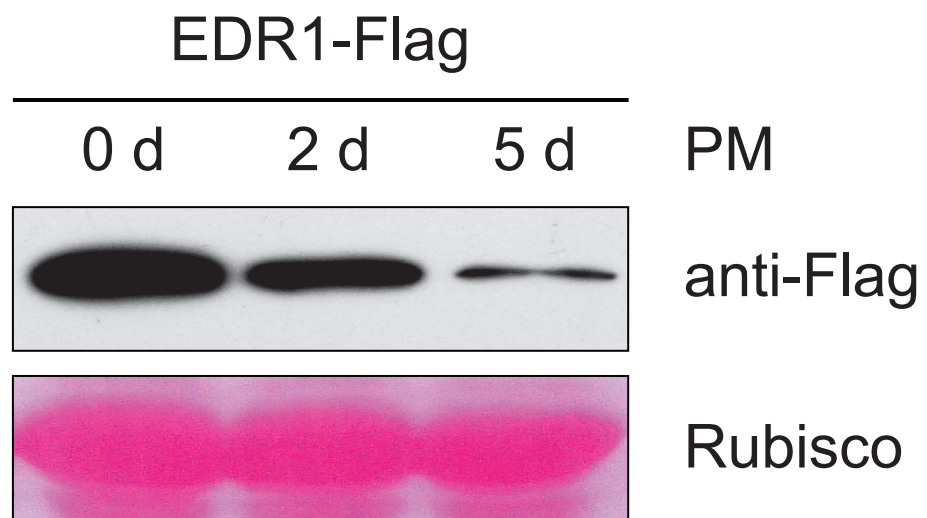

Supplement: Figure S5 — EDR1 protein level is decreased after pathogen infection. EDR1-Flag transgenic plants were infected by G. cichoracearum for 0 d, 2 d and 5 d, respectively. The infected leaves were collected at each time point and the proteins were extracted for immunoblotting using anti-Flag antibody. The experiment was repeated for three times with similar results. The large subunit of Rubisco is shown as a protein loading control. (PDF) [file pgen.1004389.s005.pdf]

Figure S6

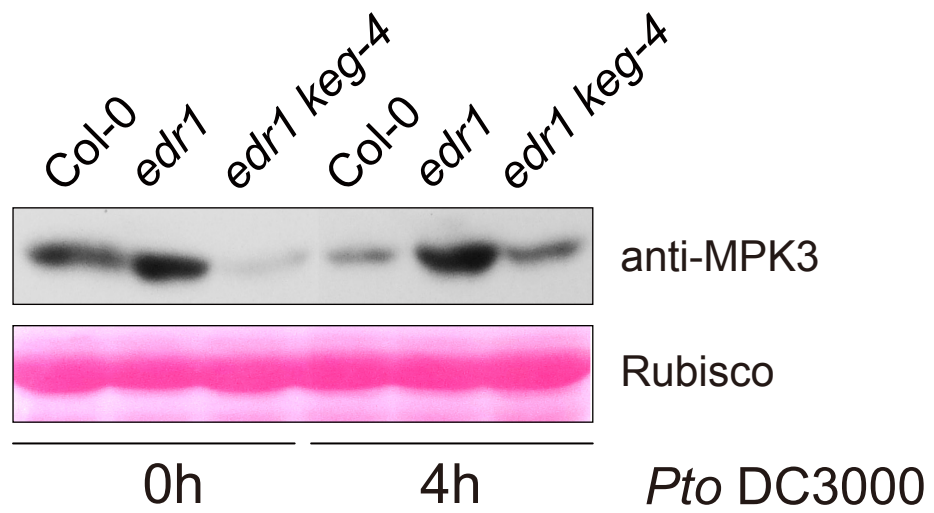

Supplement: Figure S6 — The keg-4 mutation inhibits the elevated protein levels of MPK3 in edr1. Col-0, edr1 and edr1 keg-4 mutants were infected with Pto DC3000. Proteins were extracted and immunoblots were performed using anti-MPK3 antibody. The large subunit of Rubisco is shown as a protein loading control. (PDF) [file pgen.1004389.s006.pdf]

Figure S7

A

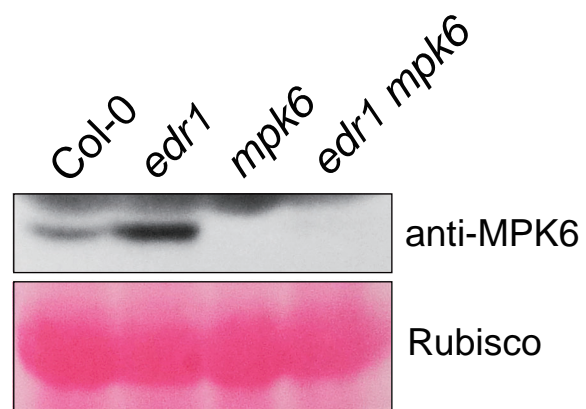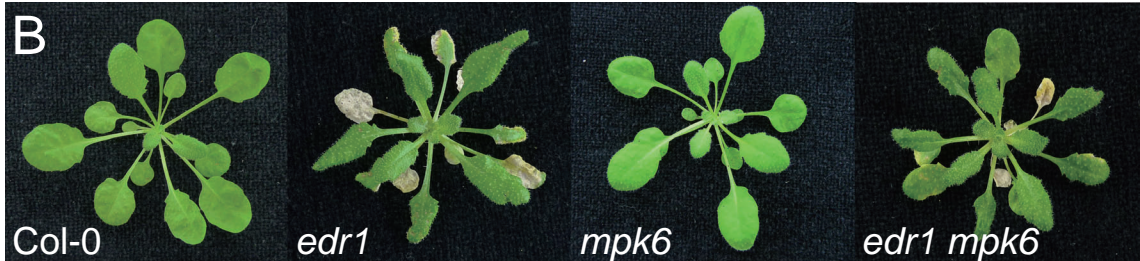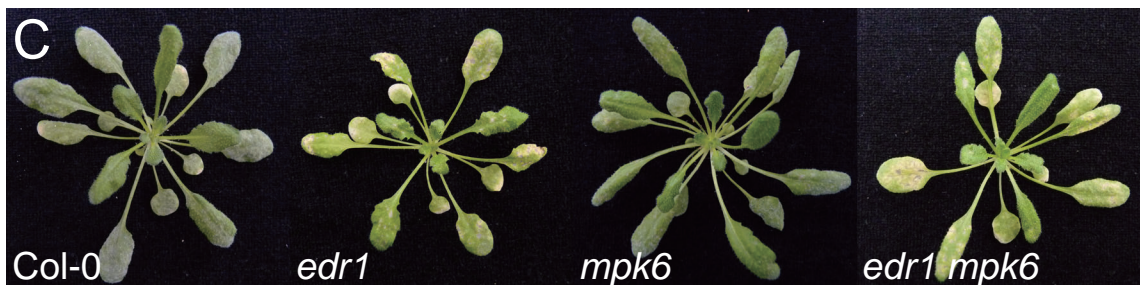

Supplement: Figure S7 — Analysis of edr1 mpk6-3 double mutant. (A) Immunoblotting was performed for Col-0, edr1, mpk6-3 and edr1 mpk6-3 using specific anti-MPK6 antibody. The large subunit of Rubisco is shown as a protein loading control. (B) Col-0, edr1, mpk6-3 and edr1 mpk6-3 were grown in the greenhouse at 22°C and a 9 h light/15 h dark cycle. Pictures were taken after 5 weeks growth. (C) Col-0, edr1, mpk6-3 and edr1 mpk6-3 were infected by G. cichoracearum. Pictures were taken at 7 dpi. (PDF) [file pgen.1004389.s007.pdf]

Figure S8

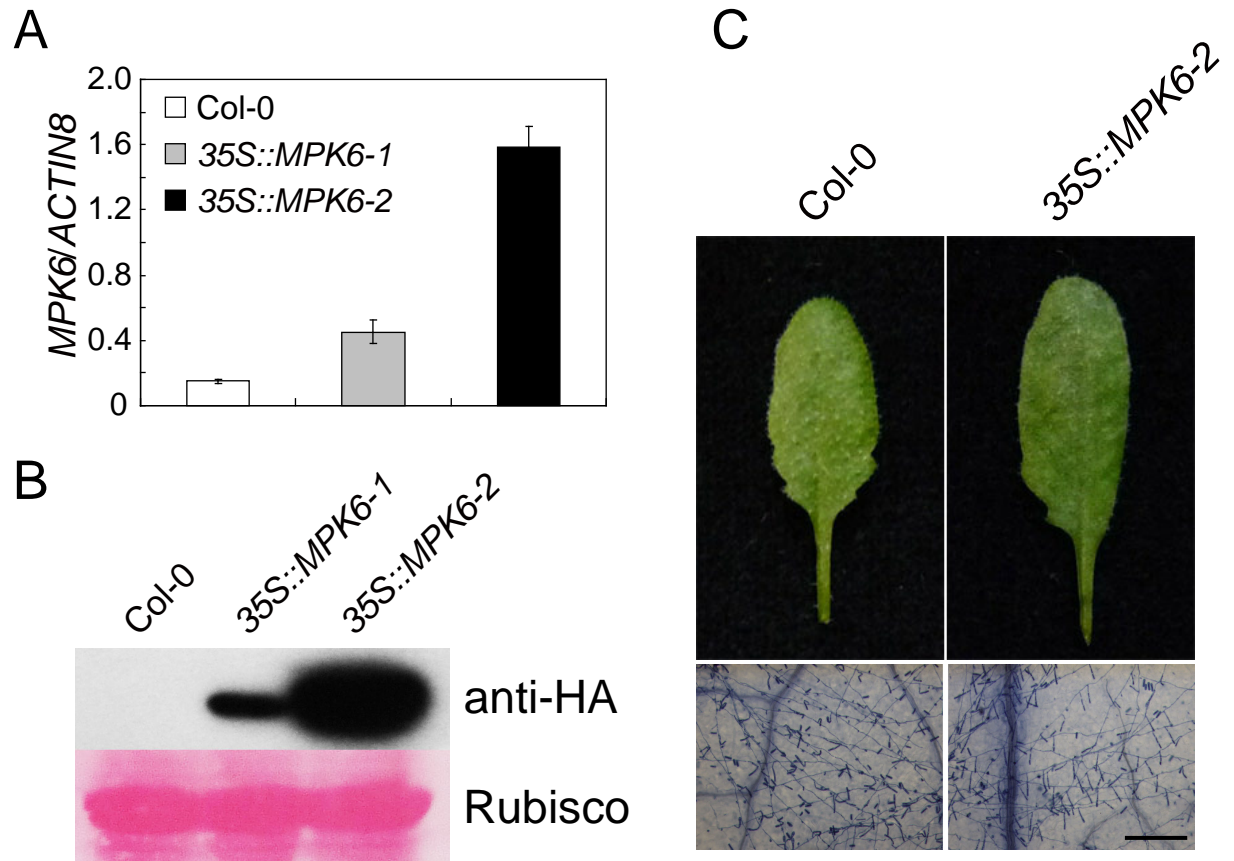

Supplement: Figure S8 — Over-expression of MPK6 in Arabidopsis. (A) Quantitative real-time RT-PCR was performed for Col-0 and MPK6 transgenic plants using MPK6 specific primers. ACTIN8 was used as the internal control. Error bars represent the standard deviation of three biological replicates. (B) Immunoblot was performed for MPK6 transgenic plants using anti-HA antibody. The large subunit of Rubisco is shown as a protein loading control. (C) Col-0 and over-expression line Col-0::MPK6-2 were inoculated with G. cichoracearum. Pictures (Top) were taken at 7 dpi. The inoculated leaves were stained by trypan blue (Bottom). Bar = 0.2 mm. (PDF) [file pgen.1004389.s008.pdf]

Figure S9

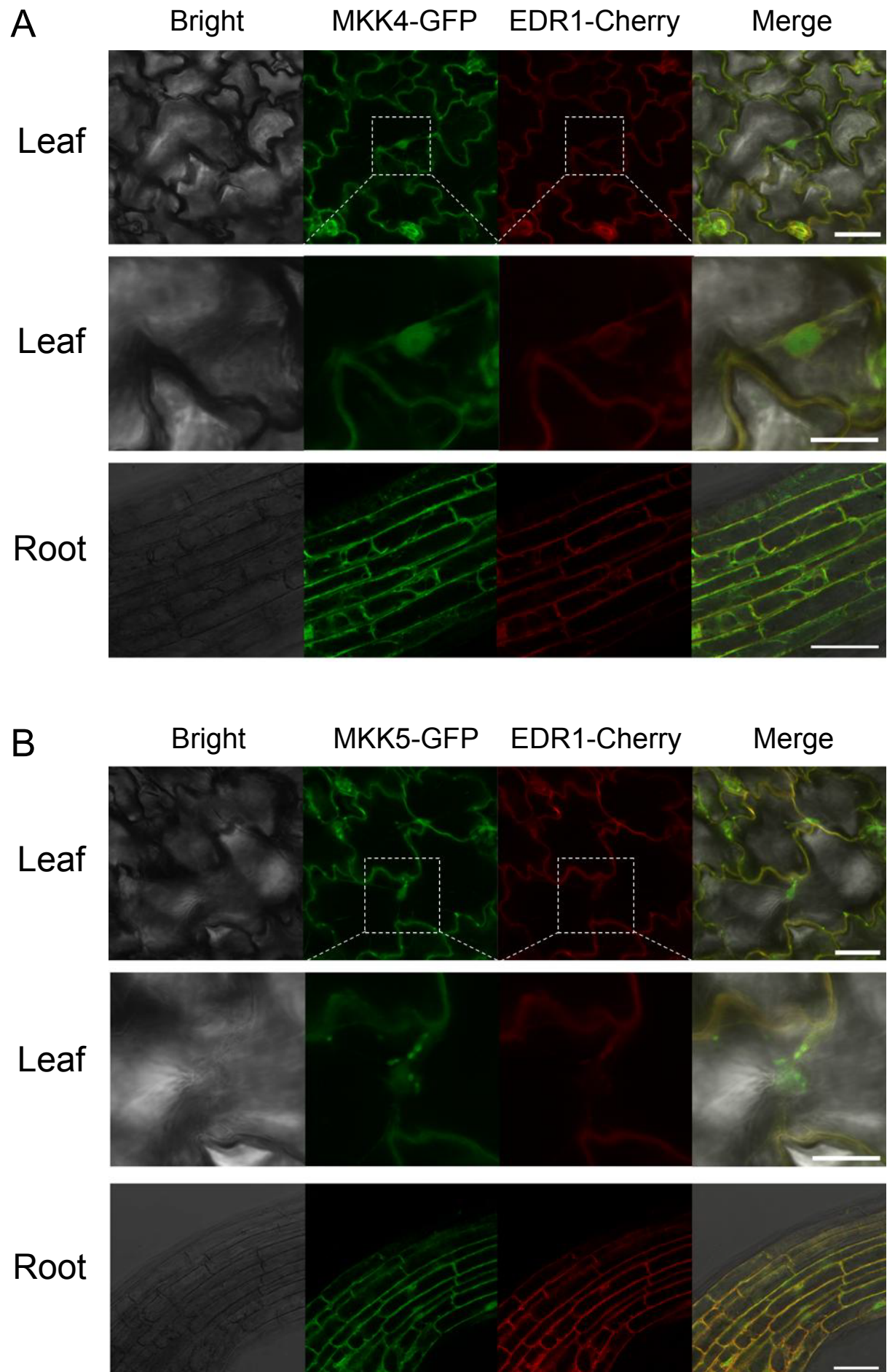

Supplement: Figure S9 — Co-localization of EDR1 and MKK4/MKK5 in Arabidopsis. The subcellular localization of MKK4-GFP and EDR1-Cherry (A) or MKK5-GFP and EDR1-Cherry (B) in leaves and roots were examined by confocal microscopy. The middle image represents higher magnification of the upper picture. Bar = 50 µm. (PDF) [file pgen.1004389.s009.pdf]

Figure S10

A

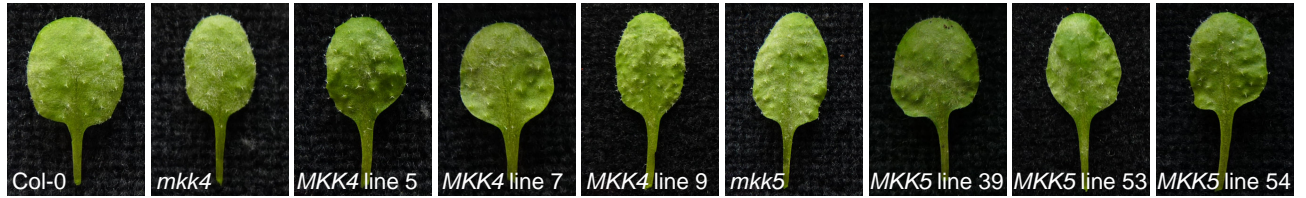

B

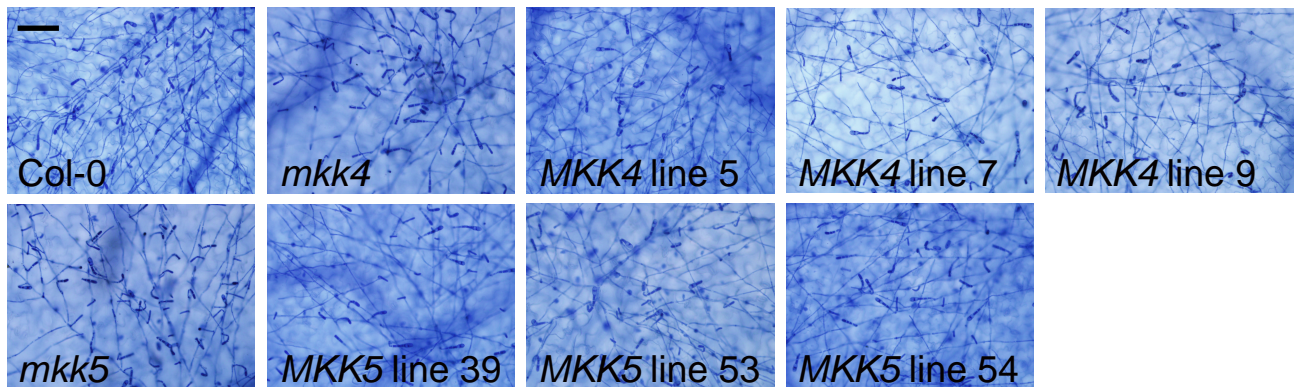

C

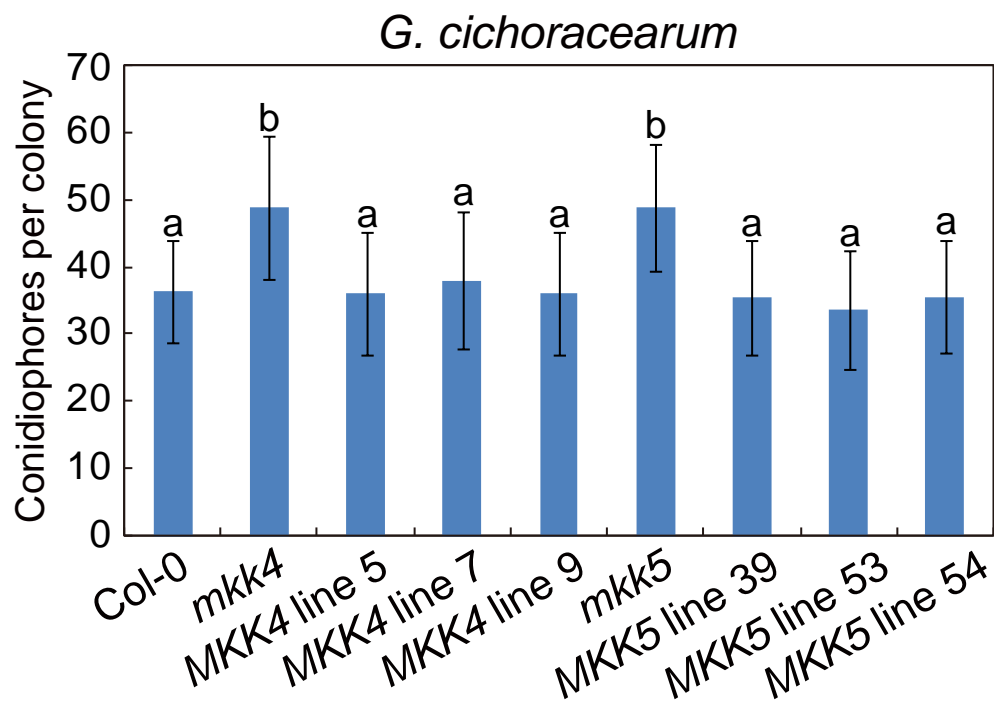

Supplement: Figure S10 — A genomic clone of MKK4 or MKK5 complemented enhanced powdery mildew susceptibility in mkk4 or mkk5. (A) Plants were infected by G. cichoracearum, and pictures were taken at 7 dpi. (B) Leaves infected with G. cichoracearum at 7 dpi were stained by trypan blue. Bar = 0.1 mm. (C) Fungal growth was assessed at 5 dpi by counting the number of conidiophores per colony. Error bars represent the standard deviation (n>30). Statistically significant differences were indicated by different letters (P<0.05, one-way ANOVA). Three independent transgenic lines of mkk4 (containing a MKK4 genomic clone, MKK4 line 5, 7 and 9) and mkk5 (containing a MKK5 genomic clone, MKK5 line 39, 53 and 54) were included. (PDF) [file pgen.1004389.s010.pdf]

Figure S11

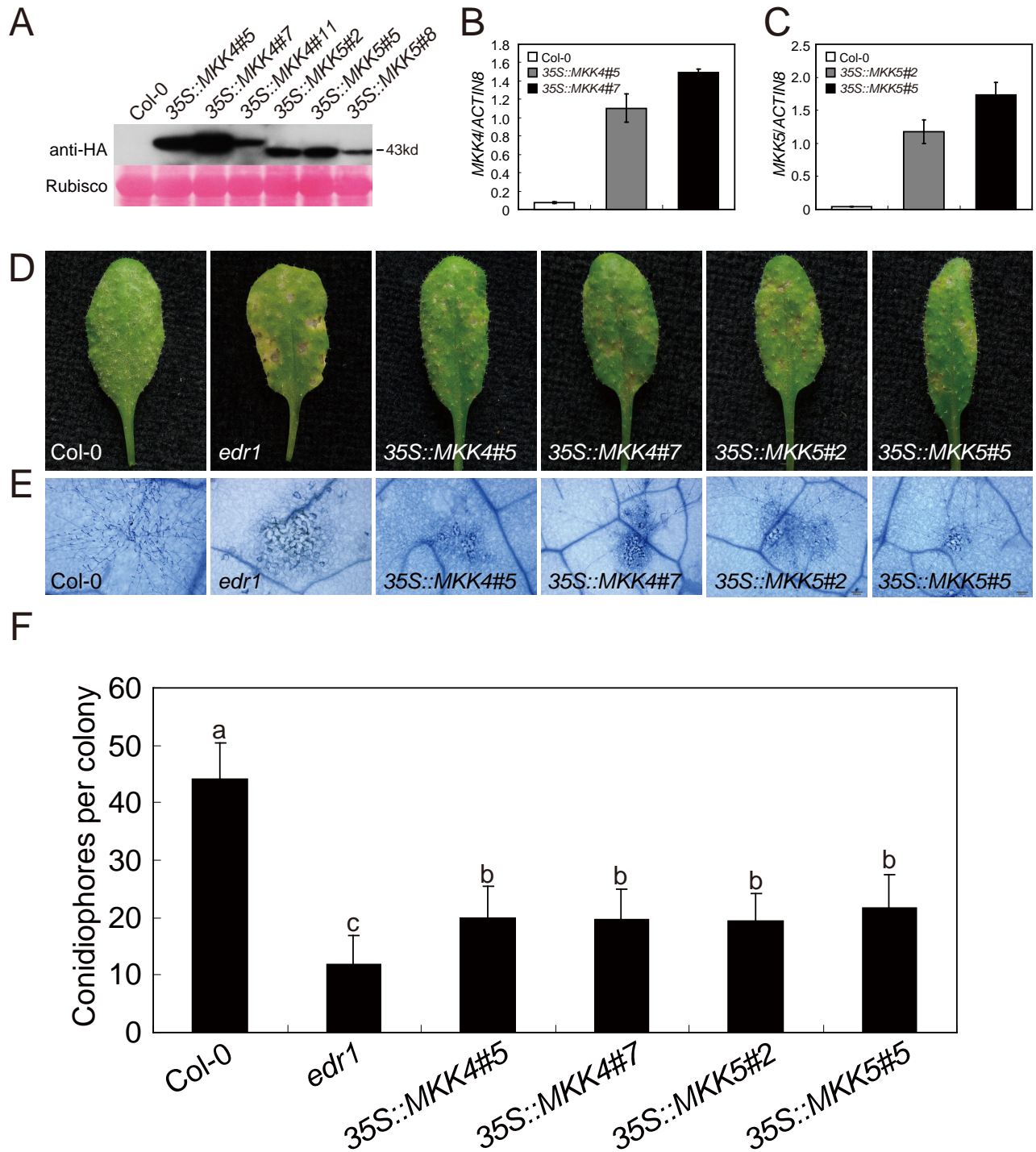

Supplement: Figure S11 — Over-expression of MKK4 and MKK5 led to edr1-like enhanced resistance to powdery mildew and mildew-induced cell death. (A) Immunoblot assay was performed for MKK4 and MKK5 transgenic plants using anti-HA antibody. Rubisco is shown as a protein loading control. (B–C) Quantitative real-time RT-PCRs were performed for MKK4 and MKK5 transgenic plants using MKK4 and MKK5 specific primers, respectively. ACTIN8 was used as an internal control. Error bars represent the standard deviation of three biological replicates. (D) Col-0, edr1 and transgenic plants 35S::MKK4 and 35S::MKK5 were infected with G. cichoracearum. Pictures were taken at 7 dpi. (E) Powdery mildew infected leaves at 7 dpi were stained by trypan blue. Pictures were taken by microscopy. Bar = 0.2 mm. (F) Fungal growth was assessed by counting the number of conidiophores per colony at 5 dpi. At least 30 colonies were counted for each sample. Error bars represent the standard deviation. Different letters represent statistically significant differences (P<0.05, one-way ANOVA). (PDF) [file pgen.1004389.s011.pdf]
